# Reversible posterior encephalopathy syndrome in a 10-year-old child

**DOI:** 10.1590/2175-8239-JBN-2018-0111

**Published:** 2018-09-21

**Authors:** Eve Grillo Carvalho, Henrique Guarino Colli Peluso, Lorena Luana Batista, Cissa Santos Moreira, Juliana Suzano Moraes Protti, Maria Cristina Bento Soares, Aline de Freitas Suassuna Autran, Amanda Rocha Soares Almeida, Denise Cristina Rodrigues, Lívia Verônica Grillo Romano Bernardes, Luciana Pimenta de Paula

**Affiliations:** 1 Universidade Federal de Viçosa Departamento de Medicina e Enfermagem ViçosaMG Brasil Universidade Federal de Viçosa, Departamento de Medicina e Enfermagem (DEM), Viçosa, MG, Brasil.

**Keywords:** Posterior Leukoencephalopathy Syndrome, Pediatrics, Nephrology, Neurology

## Abstract

**Introduction:**

The posterior reversible encephalopathy (PRES) syndrome encompasses a set of clinical-radiological findings associated with severe systemic arterial hypertension. This case report proposes to discuss the identification, diagnosis, and management of PRES in the pediatric population.

**Case presentation:**

Female patient, 10 years old, admitted to the emergency room with complaint of oliguria and generalized edema. At the initial physical exam, the only alteration present was anasarca. The diagnostic investigation revealed nephrotic syndrome, and clinical treatment was started. She evolved on the 8th day of hospitalization with peak hypertension, sudden visual loss, reduced level of consciousness, nystagmus, and focal seizures requiring intubation. She was transferred to the Intensive Care Unit, with neurological improvement, after the established therapy. CT scan revealed a discrete hypodense area in the white matter of the occipital lobe and anteroposterior groove asymmetry, compatible with PRES.

**Discussion:**

PRES is due to vasogenic cerebral edema of acute or subacute installation. Symptoms include headache and altered consciousness, stupor, coma, neurological deficits, seizures and cortical blindness. Nephropathies are the main cause of PRES in pediatrics. Magnetic resonance imaging with diffusion of molecules is the gold standard for diagnosis. The initial treatment objectives are the reduction of blood pressure, antiepileptic therapy, correction of hydroelectrolytic and acid-base disorders and management of intracranial hypertension.

**Conclusion:**

PRES is associated with acute hypertension. Early diagnosis and proper management may determine a better prognosis and minimize the severity of the clinical course.

## INTRODUCTION

The posterior reversible encephalopathy syndrome (PRES) involves a set of clinical and radiological findings characterized by a variable spectrum in severity of headache, nausea, vomiting, visual disturbances, focal neurological deficits, and seizures associated with severe systemic arterial hypertension^1^. The actual incidence of PRES remains unknown^2^, so the present article proposes to describe the clinical and radiological presentation of a PRES case in a pediatric patient, as well as to discuss the diagnosis and management of this condition.

## CASE PRESENTATION

A 10-year-old girl with a pathological history of relapsing nephrotic syndrome due to probable minimal change disease was admitted to the hospital reporting lower limb edema that started 15 days before, after weaning from corticosteroid therapy, evolving to anasarca in the last 5 days, in addition to reduction of urinary volume, coryza and dry cough. Physical exam revealed anasarca, intact neurological status, Glasgow scale 15, isochoric and light-reactive pupils, no meningeal signs or focal deficits. The girl was communicative, with discreet mucosa and cutaneous pallor, hemodynamically stable, normotensive for age and height (blood pressure in right upper limb 90 x 60 mmHg) weighing 33 kg and with estimated dry weight of 27 kg.

The initial exams identified significant hypoalbuminemia (2.1 g/dL), proteinuria (4 + / 4+), absence of azotemia (urea 36 mg/dL and creatinine 0.55 mg/dL), normal hemogram, negative anti-streptolysin O antibodies, and C-reactive protein of 3.0 mg/L. She was hospitalized with a diagnostic hypothesis of decompensated glomerulopathy, probably triggered by a nonspecific viral infection. Subsequent exams revealed nephrotic proteinuria (3506 mg in 24 hours), urine volume of 400 mL in 24 hours, equivalent to 0.62 mL/kg of estimated dry weight, reduced albumin/globulin ratio (1.0), normal kidney ultrasonography, dyslipidemia (total cholesterol 553 mg/dL and triglycerides 375 mg/dL), and normal ionogram, negative urine culture and blood culture.

Treatment was instituted with albumin (1.5 mg/kg/day for 2 days), prednisone (10 mg/day), and furosemide (40 mg/day). The dose of furosemide was increased up to 60 mg/kg/day and associated with spironolactone 50 mg/day until there was satisfactory clinical response of weight loss, significant reduction of edem, and diuresis with a flow rate greater than 1 mL/kg/hr.

On the 8th day of hospitalization, the patient had a severe hypertensive peak (180 x 100 mmHg - above the 99th percentile for age and height), being medicated with oral captopril (2 doses of 25 mg each) and intravenous hydralazine (2 doses of 0.2 mg/kg) with no success and persistence of blood pressure around 150 x 100 mmHg. Continuous monitoring and oxygen therapy were installed via nasal catheter at 2 L/min due to saturation drop in ambient air to 80%. The patient quickly developed severe holocranial headache, horizontal nystagmus, focal seizures, sudden bilateral loss of visual acuity, and reduced level of consciousness, with Glasgow ranging from 6 to 8.

Due to the rapid deterioration of the mental state and the maintenance of arterial hypertension despite the established therapy, the patient was referred to the hospital's intensive care unit (ICU), requiring a rapid intubation sequence and initiating continuous infusion of midazolam (beginning with 0.5 mcg/kg/min). After sedation, there was hemodynamic and neurological stabilization, with reduction of blood pressure ​​without the need for intravenous vasodilators and other antiepileptic drugs. She was transferred on that day in a mobile ICU to a hospital equipped with a pediatric ICU. Computed tomography performed there revealed an area of discrete hypodensity, suggestive of edema, in the white matter of the occipital lobe and anteroposterior groove asymmetry, compatible with PRES syndrome.

## DISCUSSION

The PRES syndrome is triggered by cerebral edema of vasogenic origin, typically of acute installation. Signs and symptoms include headache, altered state of consciousness leading to stupor and coma, focal neurological deficits, seizures, nausea, vomiting, mental confusion, and visual changes resulting from involvement of the occipital cortex^1^^-^4.

**Figure 1:**
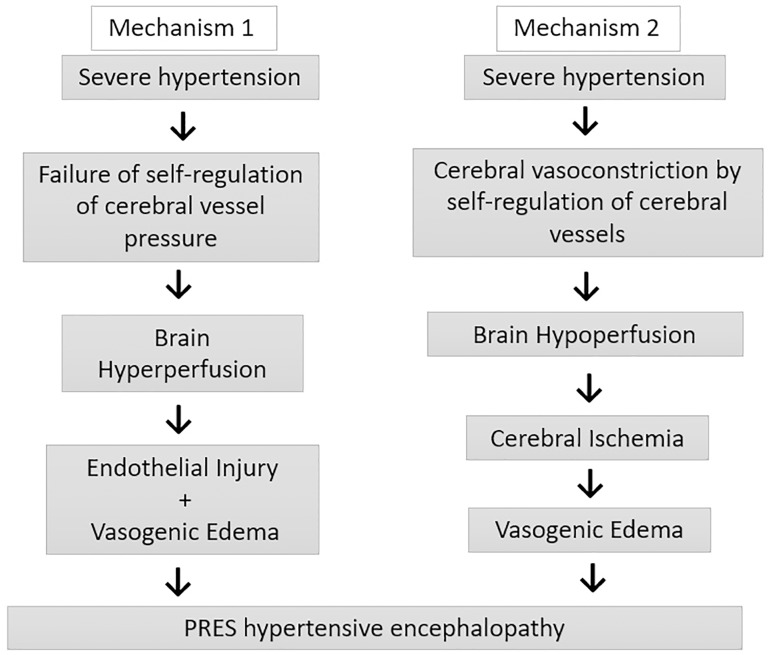
Physiopathology of Hypertensive Encephalopathy in PRES.

Pathophysiology remains controversial, and two mechanisms have been proposed. The first one suggests that severe hypertension exceeds the capacity of blood pressure self-regulation of cerebral vessels, resulting in cerebral hyperperfusion, endothelial injury, and vasogenic edema. The second theory proposes that excessive cerebral vasoconstriction due to these mechanisms of self-regulation leads to hypoperfusion and cerebral ischemia, with subsequent formation of vasogenic edema^5^. These mechanisms are outlined in Figure 1.

It is estimated that systemic arterial hypertension (SAH) has a prevalence of approximately 25% in the adult Brazilian population^6^ and the number falls to around 1% in the pediatric population. In all age groups SAH is associated to overweight / obesity and, especially in children younger than 6 years, to parenchymal and glomerular renal diseases that present with nephritic or nephrotic syndrome, renal artery stenosis or obstruction, and coarctation of the aorta in most cases^7^. This points to nephropathies as the major cause of PRES in childhood and explains the relative rarity of reported cases of PRES in pediatric patients when compared to the adult population.

The major radiological finding is cerebral edema, and diffusion magnetic resonance imaging (MRI) is the current gold standard, since it allows the differentiation of cytotoxic edema from the vasogenic edema suggestive of PRES^8^^,^^9^. Fugate et al. analyzed imaging studies of 120 cases and concluded that the most commonly affected region was the parietal-occipital, predominantly the subcortical area^10^.

**Figure 2:**
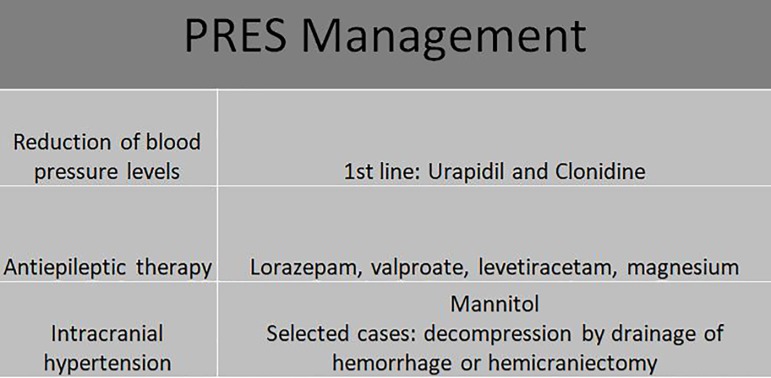
Therapeutic Management of PRES.

The adequate management of the hypertensive crisis in the context of the neurovascular emergency, with risk of imminent organ damage, aims to reduce blood pressure levels by 20-25% in two hours, and the use of urapidil and clonidine as the first line of treatment is indicated. For antiepileptic therapy, benzodiazepines, valproate, levetiracetam, and magnesium are effective therapeutic alternatives. The levels of serum magnesium should be evaluated periodically if it is used in therapy. Mannitol may be considered for the reduction of intracranial hypertension and, only in selected cases, drainage decompression for eventual hemorrhage or even hemicraniectomy^11^. The clinical management is outlined in Figure 2.

As to the prognosis, 25-45% of the patients present persistent radiological findings and 10-25% present neurological deficits without complete recovery. Up to 8% of patients will require chronic treatment for epilepsy. The recurrence rate is 5 to 15% over 2 years^11^.

## CONCLUSION

PRES results from the failure of the cerebrovascular auto-regulation mechanisms at high blood pressures. Secondary hypertension in children is commonly related to renal changes, suggesting that these are the main etiology of PRES in childhood. Early diagnosis and proper management can determine a better prognosis and minimize the severity of its clinical course.
